# Supplementary material for: Electropositive Membrane Prepared via a Simple Dipping Process: Exploiting Electrostatic Attraction Using Electrospun SiO2/PVDF Membranes with Electronegative SiO2 Shell
Source: Polymers (Basel). 2023 May 11;15(10):2270. doi: 10.3390/polym15102270 (PMC10223967; doi:10.3390/polym15102270)
Supplement: Supplementary file 1 [file polymers-15-02270-s001.zip › polymers-2371903-supplementary.pdf]

Supporting Information

**samples**	Before attachment (mg)	After attachment (mg)	Wt. difference (mg)	Increase of Wt (%)
1	8.0	9.0	1.0	12.50 %
2	9.3	11.0	1.7	18.28 %
3	9.1	10.1	1.0	10.99 %
4	9.2	10.3	1.1	11.96 %
5	8.7	10.0	1.3	14.94 %
6	10.8	12.0	1.2	11.11 %
7	10.3	11.7	1.4	13.59 %
8	9.6	10.7	1.1	11.46 %
9	10.2	11.4	1.2	11.76 %
10	10.1	11.1	1.0	9.90 %
Average 12.65 wt% boehmite was attached by membrane				

Figure S1. Attachment of boehmite onto the electrospun SiO_2/PVDF membrane
